# High Intensity Exercises in Heart Failure with Preserved Ejection Fraction

**DOI:** 10.5935/abc.20180225

**Published:** 2018-11

**Authors:** Artur Haddad Herdy, Magnus Benetti

**Affiliations:** 1 Instituto de Cardiologia de Santa Catarina, Florianópolis, SC - Brazil; 2 Universidade do Sul de Santa Catarina (UNISUL), Florianópolis, SC - Brazil; 3 Universidade do Estado de Santa Catarina (UDESC), Florianópolis, SC - Brazil

**Keywords:** Heart Failure, Stroke Volume, Obesity, Exercise, Exercise Therapy, Breating Exercises

## Introduction

Heart failure with preserved ejection fraction (HFpEF) comprises several pathologies that present with variable degrees of dyspnea, high filling pressures, structural or diastolic alterations and great limitation to exercise.^1^ HFpEF can represent up to 50% of cases of hospital admissions due to decompensated heart failure (HF).^2^

Hypertension and obesity are conditions frequently associated with HFpEF and the adequate management of these two pathologies are essential for the treatment of this syndrome. One of the main characteristics of patients with HFpEF is the intolerance to exercise at different degrees and through diverse mechanisms.^3^

Exercises are among the main therapeutic strategies for the treatment of heart failure with reduced ejection fraction (HFrEF) and HFpEF, being important agents in decreasing the morbidity and mortality of these patients.^4^^-^^6^

Among the benefits of aerobic training in patients with HFpEF, we can highlight the improvement in endothelial function and arterial stiffness, contributing to the improvement of cardiovascular dynamics and symptoms.^7^ The physical training programs offered to patients with HF in cardiac rehabilitation services involve primarily aerobic exercises supplemented by resistant exercises, stretching and, in some cases, respiratory exercises.^1^

Aerobic exercises can be continuous, of moderate intensity or intercalating high and low-intensity efforts. High-intensity interval training (HIIT) is currently one of the most effective methods for improving cardiorespiratory and metabolic function. HIIT involves repeated activities, from short to long ones, of high-intensity exercises combined with periods of active or passive recovery.^8^ Kiviniemi et al. have recently reported that HIIT is superior to traditional continuous aerobic training in improving cardiac autonomic function and suggested that the effect verified on post-HIIT autonomic function was related to improved baroreflex modulation and vagal control.^9^

There are several potential adaptations that explain the positive changes induced by HIIT on the autonomic cardiac function. One of the potential mechanisms related to HIIT-induced improvement in cardiac vagal tone may be angiotensin II, which inhibits cardiac vagal activity. Sedentary or physically inactive individuals have higher plasma renin activity when compared to those who are physically active. Exercise causes angiotensin II suppression, which can, to some extent, mediate the improvement in cardiac vagal tone.^10^ Studies have also suggested that HIIT induces increased baroreflex sensitivity and reduces arterial stiffness.^11^

### Comments about the current study

In this interesting study, designed for the assessment of the acute effects of a single session of high-intensity interval training, Lima et al.^12^ studied post-training changes in blood pressure (BP) and endothelial function in 16 patients with HFpEF. As main results, it was possible to demonstrate a significant increase in the brachial artery diameter with a corresponding reduction in systolic BP. These findings indicate the potential benefit of this type of training for patients with HFpEF, with an improvement in blood pressure levels and, possibly, a beneficial effect on ventricular function.

Although the authors did not find any significant changes in the flow-mediated dilation index, questions have been raised about the real importance and interpretation of this measurement.^13^ The BP reduction after the exercise sessions tends to last for hours, acting as powerful adjuvants to the vasodilation effects of antihypertensive drugs, which are commonly used in HFpEF. BP control is among the main goals for symptom improvement in HFpEF, and exercises are crucial to attain this goal and improve diastolic function.^14^

## Limitations and conclusions

This experiment used a single training group, without a control group for better definition of the effects and lower chance of bias when assessing the results. Although the number of patients was small, the positive results encourage better designed future researches, with a larger number of individuals to define the role of this training modality in HFpEF. These patients have an expressive limitation to exercises and strategies that improve BP and diastolic function show great potential for benefits in functional class improvement and, likely, in morbimortality reduction.

## References

[1] Comitê Coordenador da Diretriz de Insuficiência
Cardíaca (2018). Diretriz Brasileira de Insuficiência Cardíaca
Crônica e Aguda. Arq Bras Cardiol.

[2] Yancy CW, Lopatin M, Stevenson LW, De Marco T, Fonarow GC, ADHERE Scientific Advisory Committee and Investigators (2006). Clinical presentation, management, and in-hospital outcomes of
patients admitted with acute decompensated heart failure with preserved
systolic function: a report from the Acute Decompensated Heart Failure
National Registry (ADHERE) Database. J Am Coll Cardiol.

[3] Houstis NE, Eisman AS, Pappagianopoulos PP, Wooster L, Bailey CS, Wagner PD (2018). Exercise intolerance in heart failure with preserved ejection
fraction: diagnosing and ranking its causes using personalized O2 pathway
analysis. Circulation.

[4] Piepoli MF, Davos C, Francis DP, Coats AJ, ExTraMATCH Collaborative (2004). Exercise training meta-analysis of trials in patients with
chronic heart failure. BMJ.

[5] O'Connor CM, Whellan DJ, Lee KL, Keteyian SJ, Cooper LS, Ellis SJ, HF-ACTION Investigators (2009). Efficacy and safety of exercise training in patients with chronic
heart failure: HF-ACTION randomized controlled trial. JAMA.

[6] Edelmann F, Gelbrich G, Düngen HD, Fröhling S, Wachter R, Stahrenberg R (2011). Exercise training improves exercise capacity and diastolic
function in patients with heart failure with preserved ejection fraction:
results of the Ex-DHF (Exercise training in Diastolic Heart Failure) pilot
study. J Am Coll Cardiol.

[7] Kitzman DW, Brubaker PH, Herrington DM, Morgan TM, Stewart KP, Hundley WG (2013). Effect of endurance exercise training on endothelial function and
arterial stiffness in older patients with heart failure and preserved
ejection fraction: a randomized, controlled, single-blind
trial. J Am Coll Cardiol.

[8] Tschakert G, Hofmann P (2013). High-Intensity Intermittent Exercise: Methodological and
Physiological Aspects. Int J Sports Physiol Perform.

[9] Kiviniemi A, Tulppo M, Eskelinen J, Savolainen A, Kapanen J, Heinonen I (2014). Cardiac autonomic function and high-intensity interval training
in middle-age men. Med Sci Sports Exerc.

[10] Buch AN, Coote JH, Townend JN (2002). Mortality, cardiac vagal control and physical training - What's
the link. Exp Physiol.

[11] Heydari M, Boutcher YN, Boutcher SH (2013). High-intensity intermittent exercise and cardiovascular and
autonomic function. Clin Auton Res.

[12] Lima JB, Silveira AD, Saffi MAL, Menezes MG, Piardi DS, Ramm LDCR (2018). Vasodilation and Reduction of Systolic Blood Pressure after One
Session of High-Intensity Interval Training in Patients With Heart Failure
with Preserved Ejection Fraction. Arq Bras Cardiol.

[13] Atkinson G, Batterham AM (2015). The clinical relevance of the percentage flow-mediated dilation
index. Curr Hypertens Rep.

[14] Pearson MJ, Mungovan SF, Smart NA (2017). Effect of exercise on diastolic functional in heart failure
patients: a systematic review and meta-analysis. Heart Fail Rev.

